# School ethos and adolescent gambling: a multilevel study of upper secondary schools in Stockholm, Sweden

**DOI:** 10.1186/s12889-020-8230-y

**Published:** 2020-01-30

**Authors:** Sara Brolin Låftman, Bitte Modin, Gabriella Olsson, Kristina Sundqvist, Johan Svensson, Peter Wennberg

**Affiliations:** 1 Department of Public Health Sciences, Centre for Health Equity Studies (CHESS), Stockholm University, SE-10691 Stockholm, Sweden; 2 Department of Psychology, Stockholm University, SE-10691 Stockholm, Sweden; 3 Department of Public Health Sciences, Stockholm University, SE-10691 Stockholm, Sweden; 4 Department of Public Health Sciences, Karolinska Institutet, SE-17177 Solna, Sweden

**Keywords:** Adolescent gambling, Problem gambling, Youth, School, Contextual

## Abstract

**Background:**

Gambling is not uncommon among adolescents, and a non-trivial minority has serious problems with gambling. Therefore, enhanced knowledge about factors that may prevent against problematic gambling among youth is needed. Prior research has shown that a strong school ethos, which can be defined as a set of attitudes and values pervading at a school, is associated with a lower inclination among students to engage in various risk behaviours. Knowledge about the link between school ethos and adolescent gambling is however scarce. The aim of the study was to investigate the association between teacher-rated school ethos and student-reported gambling and risk gambling, when controlling also for sociodemographic characteristics at the student- and the school-level.

**Methods:**

Data from two separate cross-sectional surveys were combined. The Stockholm School Survey (SSS) was performed among 5123 students (aged 17–18 years) in 46 upper secondary schools, and the Stockholm Teacher Survey (STS) was carried out among 1061 teachers in the same schools. School ethos was measured by an index based on teachers’ ratings of 12 items in the STS. Adolescent gambling and risk gambling were based on a set of single items in the SSS. Sociodemographic characteristics at the student-level were measured by student-reported information from the SSS. Information on sociodemographic characteristics at the school-level was retrieved from administrative registers. The statistical method was multilevel regression analysis. Two-level binary logistic regression models were performed.

**Results:**

The analyses showed that higher teacher ratings of the school’s ethos were associated with a lower likelihood of gambling and risk gambling among students, when adjusting also for student- and school-level sociodemographic characteristics.

**Conclusions:**

This study showed that school ethos was inversely associated with students’ inclination to engage in gambling and in risk gambling. In more general terms, the study provides evidence that schools’ values and norms as reflected by the teachers’ ratings of their school’s ethos have the potential to counteract unwanted behaviours among the students.

## Background

Offering gambling to minors is prohibited in most judicial contexts. Yet, gambling is not an uncommon activity among adolescents, and a non-trivial minority has serious problems with gambling. Turchi and Derevensky [1] have described adolescent gambling as a continuum reaching from “no gambling” at one endpoint to “problem gambling” at the other, with “social gambling” and “at-risk gambling” in between. According to a definition by Neal et al. [2], “Problem gambling is characterised by difficulties in limiting money and/or time spent on gambling which leads to adverse consequences for the gambler, others, or for the community.” (p. 3) According to a recent review of 44 studies from across the world, the prevalence of problem gambling among youth has been reported to vary between 0.2 and 12.3% [3]. The latest report from the European School Survey Project on Alcohol and Other Drugs (ESPAD) indicates that Swedish students gamble slightly less than European students on average. Of the Swedish students aged 16 years, 13% had gambled at least once during the past 12 months, while the European average was 14%. More frequent gambling, i.e. twice or more a month during the past 12 months, was reported by 5% of the Swedish students, whereas the European average was 7% [4].

### Background and previous research

Prior research on adolescent gambling has largely concentrated on risk and preventive factors at the individual- and the family-level. There is a clear and consistent gender difference in that both gambling and risk gambling are more common among boys than among girls [1, 3–7]. Other risk factors at the individual-level include personality traits such as impulsivity and sensation-seeking, but also anxiety, depression, attention deficit hyperactivity disorder (ADHD), substance use, and delinquency [6, 8]. With regards to conditions in the family, earlier research has shown that parental gambling is a risk factor for adolescent gambling [8, 9], whereas family cohesion, family support and parental monitoring are inversely associated with problematic gambling among adolescents [5, 8–10].

Yet, adolescent behaviour is not only shaped by individual and family-related conditions, but also by other social contexts in which they are embedded. One important arena in adolescents’ lives is the school, where different types of behaviours are shaped in relationships with peers but also through values and norms conveyed by the school as such. As noted by Lee et al. [6], research on the school context in relation to gambling is limited. Yet, some previous studies have identified certain school-related conditions as important predictors of problematic gambling among the students. At the individual level, poor school performance has been shown to be linked with an increased risk of problem gambling [8], whereas a negative association has been reported for adolescents’ perception of school connectedness and problematic gambling [5]. Elgar et al. [10] showed that students’ perception of teacher support and of classmate support were inversely associated with excessive gambling.

With regards to conditions measured at the school-level, Lee et al. [6] showed, using school survey data from Maryland, US, that schools’ suspension rate and the proportion of African American students had weak, negative associations with gambling but not with gambling problems. Elgar et al. [10] demonstrated that relative deprivation, measured as being worse off than one’s classmates in terms of family wealth, was associated with an increase in the rate of excessive gambling. To the best of our knowledge, however, few studies have investigated if more qualitative aspects of schools’ organisational functioning are related to students’ inclination to engage in gambling and in risk gambling.

Research on “school effectiveness” has focused on how organisational and environmental aspects of the school environment affect students’ academic performance and behaviour. In their work carried out in schools in London in the 1970s, Michael Rutter and his colleagues found that students who attended schools with certain qualities performed better, irrespective of their social background [11]. Specific features of school effectiveness include positive student-teacher relationships, clear goals and expectations for behaviour, high expectations and feedback to the students regarding their school performance, a strong school ethos [11], as well as a strong school leadership [12].

The concept of school ethos is a central component of effective schools and can be defined as the set of norms, values, attitudes and behaviours that prevail at a school [11]. A closely related concept is school climate, but whereas school climate is often captured by students’ responses about the school environment which have been aggregated to the school level [13–15] and thus reflects the students’ general experiences of their school environment, the concept of school ethos is more closely related to the school leadership’s efforts to promote a beneficial learning environment and to counteract unwanted behaviour [16].

A strong school ethos has been shown to be linked with higher student performance [11, 17, 18]. In addition, research has shown that students attending schools with a strong ethos are less inclined to engage in health risk behaviours [19–21] and in other unwanted behaviours such as bullying perpetration [16] and truancy [22]. Yet, to the best of our knowledge, studies of the potential link between school ethos and adolescent gambling and risk gambling are lacking. Nevertheless, there are reasons to believe that school ethos may affect students’ inclination also to engage in gambling as well as risk gambling. One potential mechanism may be school performance and motivation. A strong school ethos enhances school achievement and motivation, and it is reasonable to assume that students who are study-oriented and who have high aspirations are less prone to engage in risk behaviours than their less academically motivated peers. Another possible mechanism may be perceived teacher caring. Students attending schools characterised by a strong school ethos have been shown to report higher perceived teacher caring compared with students in schools with a weaker ethos [23]. Positive relations with teachers have in turn been shown to function protectively against risk behaviours such as smoking, drunkenness, and cannabis use [24]. Still another pathway may be via students’ future orientation. A strong school ethos has been shown to be connected with a more optimistic future orientation among the students [25]. Feelings of hopelessness and a sense of a lack of future prospects may in turn make individuals more prone to risk behaviours. Indeed, earlier studies have shown that adolescents with a pessimistic future orientation are more likely to engage in different health risk behaviours [26], including risk gambling [27].

### Aim of the study

The aim of the current study was to investigate the association between teacher-rated school ethos and students’ self-reports of gambling and of risk gambling, when controlling also for sociodemographic characteristics at the student- and the school-level.

## Methods

### Data material

The data material was based on merged information from two cross-sectional surveys performed in 2016: the Stockholm School Survey (SSS) and the Stockholm Teacher Survey (STS). In addition, we linked administrative register information on schools from the Swedish National Agency for Education on the schools’ proportion of students whose parents have a post-secondary education and proportion of students with a foreign background.

The SSS is a classroom survey performed biannually among students in the ninth grade of primary school (ages 15–16 years) and in the second grade of upper secondary school (ages 17–18 years) in all public schools and in a large number of independent schools in Stockholm municipality. The questionnaire includes questions on risk behaviours including alcohol consumption, drug use, smoking and gambling, but also covers aspects such as the school environment and psychological health. In 2016, the response rate was approximately 78% [28].

The STS is a web survey carried out among teachers in the same schools that participated in the SSS. The main purpose of the survey was to collect information about school characteristics, such as teachers’ ratings of the school ethos and of their work environment, and to link these assessments to data collected among students in the SSS. In the STS of 2016, the response rate among upper secondary school teachers was 58% (*n* = 1414).

The present study used data from three sources: the SSS of 2016 collected among students in the second grade of upper secondary school, school-level information collected from upper secondary school teachers in the STS of 2016, and information on schools from administrative registers retrieved from the Swedish National Agency for Education. Information from these three sources was available for 46 upper secondary schools, covering survey information from 5604 students and 1061 teachers. (The high number of responding teachers in relation to the number of responding students is due to the fact that the STS targeted all the teachers in the schools, whereas the SSS was completed only by students in the second grade.) In the present study, the aspects of interest were school ethos (measured by information from the STS) and gambling and risk gambling (measured by information from the SSS). In addition, we controlled for sociodemographic characteristics at the student-level, i.e., gender, family structure, parental university education, parental unemployment and migration background (measured by information from the SSS). The level of teacher-rated school ethos has been shown to vary by schools’ sociodemographic composition [18], and in order to isolate the “effect” of school ethos on gambling and on risk gambling as far as possible, we also included two control variables at the school-level: the proportion of students with parents with post-secondary education and the proportion of students with a foreign background (measured by information from the Swedish National Agency for Education).

For the analyses, 481 students were excluded due to item non-response on gambling or on the control variables, leading to a study sample of 5123 students. More information on the data material is provided in the Technical Report [29].

### Variables

#### Dependent variables (student-level)

*Gambling* was assessed by the question “Have you bought lottery tickets or gambled for money at any time during the last 12 months?” including the specification “*(Scratch ticket, game show lottery, casino, poker, betting on football, horses or the like, also on the Internet)*”*.* The response categories were “No” and “Yes”.

*Risk gambling* was captured by three questions that were asked to those students who had responded that they had been gambling in the past 12 months: “How many times during the last 12 months have you”: a) “Tried to reduce your gambling?”, b) “Felt restless and irritated if you haven’t been able to gamble, and c) “Lied about how much you’ve gambled?” For each of these, the students were asked to mark one of the response categories “Never”, “1–2 times”, and “3 times or more”. For every question, we dichotomised the answers and coded students responding “Never” into one category and those responding once or more often into the other category. A dichotomous measure of risk gambling was constructed by classifying students who answered “at least once” to any of the three questions as engaging in risk gambling. The validity of the measure has been examined in a previous study, which reported internal consistency (Cronbach’s alpha = 0.66) and unidimensionality of the three items [30]. The measure of risk gambling was intended to capture gambling with (at least) some negative consequences [31]. According to our operationalization, the prevalence of risk gambling was about 3.3%. This is in line with other Swedish studies. For instance, according to figures from the Swedish longitudinal gambling study (Swelogs) of 2015, about 1% of 16–17-year-olds were classified as problem gamblers and 2.5% were at risk of problem gambling [32].

#### Independent variable (school-level)

*School ethos* was measured by an index constructed from twelve statements in the STS: a) “At this school we have a value system (“värdegrund”) which is clear to students”; b) “At this school the teachers make an effort to provide positive feedback about students’ performance”; c) “Teachers have high expectations of student performance”; d) “Teachers at this school take their time with students even if they want to discuss something other than school work”; e) “At this school we actively work on issues such as violence, bullying and harassment among students”; f) “This school provides a stimulating learning environment”; g) “The teachers at this school have a strong work ethic”; h) “The teachers work with strong enthusiasm”; i) “At this school the students are treated with respect”; j) “The teachers at this school feel confident as classroom leaders”; k) “At this school students’ motivation is a stimulating part of work” and l) “There is high staff turnover amongst teachers at this school”. The first eleven items had the following response categories: (5) “Strongly agree”; (4) “Agree”; (3) “Neither agree nor disagree”; (2) “Disagree”; and (1) “Strongly disagree”, The last item l) had a four-point scale with the response categories (1) “Agree completely”, (2) “Agree somewhat”, (3) “Disagree somewhat”, and (4) “Disagree completely”. The values from all responses were added to an index with the possible range 12–59, with higher values indicating stronger school ethos. The school ethos measure was originally developed for the STS, and has been used in prior studies based on the same data material [18, 25]. The items were formulated with the purpose of measuring teachers’ ratings of school ethos as conceptualized in the scholarship of school effectiveness [11, 16–18]. The index was developed through exploratory and confirmatory factor analysis which showed that the measure had satisfactory psychometric properties (RMSEA = 0.079; CFI = 0.931; TLI = 0.916) and high internal consistency (Cronbach’s alpha = 0.88) [29]. The mean value of the index, based on information from all the responding teachers in a school, was used as a school-level measure of school ethos. In order to assess potentially non-linear effects, the study sample was divided into categories of about equal size according to the value of school ethos, in order to capture schools with a relatively weak, intermediate, and strong teacher-rated school ethos. This strategy has been used also in previous studies using the same data material [23, 25].

#### Control variables (student-level)

*Gender* was assessed by the question “Are you a boy or a girl?”, with the response categories “Boy” and “Girl”.

*Family structure* was constructed from the question “Which people do you live with?” followed by a list of non-mutually exclusive response categories. Students who marked “Mother” and “Father” were coded as living with two parents in one household. This category was contrasted against all others.

*Parental university education* was captured by the question “What is the highest education your parents have?” followed by a set of response categories to be ticked separately for the mother and the father: “Old elementary school (“folkskola”) or compulsory school (max 9 years schooling)”, “Upper secondary school”, “University and university college”, and “Don’t know”. Since quite large proportions of students marked “Don’t know” or skipped the question (17.5% for the mother and 21.6% for the father in the study sample), we created a dichotomous variable indicating if students had marked “University and university college” for one or both parents. This group was defined as having at least one parent with university education, and was categorised against all others.

*Parental unemployment* was constructed from the question “What do your parents do?” followed by a list of response categories, to be marked separately for the mother and the father. Students who marked “Unemployed” for one or both parents were defined as having at least one unemployed parent, and were contrasted to all others.

*Migration background* was assessed by the question “How long have you lived in Sweden?” with the response categories: “All my life”, “10 years or more”, “5–9 years” and “Less than 5 years”. For the present study, the last two categories were collapsed due to small numbers.

#### Control variables (school-level)

*Proportion of students with parents with post-secondary education* was retrieved from the Swedish National Agency for Education and reported in per cent.

*Proportion of students with a foreign background* (i.e. proportion of students born outside Sweden or whose both parents were born outside of Sweden) was retrieved from the Swedish National Agency for Education and reported in per cent.

### Statistical method

Since the aim was to examine the association between one independent variable at the school-level (teacher-rated school ethos) and two dependent variables at the student-level (student-reported gambling and risk gambling), the statistical method used was multilevel modelling. Two-level binary logistic regression models were performed in Stata (version 15) using the “xtmelogit” command. Odds ratios with 95% confidence intervals are reported.

For both outcomes, we first fitted an empty model, containing no independent variables. Model(s) 1 included only student-level variables. Model(s) 2 added the school proportion of students whose parents have post-secondary education, and Model(s) 3 added the school proportion of students with a foreign background. Model(s) 4 added categorical teacher-rated school ethos. In addition, for both outcomes we performed analyses using the continuous, standardised (mean = 0, s.d. = 1) version of the school ethos measure (not presented in Tables). For all models, we report the intraclass correlation (ICC) which for binary outcomes is an approximate estimate of how much of the variance in the dependent variable that can be attributed to the higher level.

## Results

Descriptive statistics of the data are presented in Table 1. In the study sample, 13.8% of the students responded that they had been gambling at least once during the past 12 months. With regards to our measures of risk gambling, 2.1% of the students reported that they had tried to reduce their gambling, 1.7% that they had felt restless or irritated when they had not been able to gamble, and 1.4% that they had lied about their gambling. In all, 3.3% had reported at least one of the separate measures of risk gambling.

**Table 1:** Descriptives. *n* = 5123 students distributed across 46 upper secondary schools

*Student level*	n	%		
Gambling				
Gambling during the past 12 months	707	13.8		
Risk gambling				
Tried to reduce gambling	109	2.1		
Felt restless or irritated when not able to gamble	86	1.7		
Lied about gambling	69	1.4		
At least one indicator of risk gambling	169	3.3		
Gender				
Boy	2380	46.5		
Girl	2743	53.5		
Family structure				
Two parents in the same household	3280	64.0		
Other	1843	36.0		
Parental university education				
No parent	1708	33.3		
At least one parent	3415	66.7		
Parental unemployment				
No parent	4808	93.9		
At least one parent	315	6.1		
Migration background				
≥ 10 years in Sweden	4651	90.8		
< 10 years in Sweden	472	9.2		
*School level*	Mean	s.d.	Min	Max
School ethos	46.1	4.5	34.8	54.3
Weak (*n* = 1722)	41.3	2.7	34.8	44.3
Intermediate (*n* = 1743)	46.0	1.1	44.4	48.0
Strong (*n* = 1658)	51.2	1.6	48.1	54.3
School ethos (std.)	0.0	1.0	−2.5	1.8
% students with parents with post-secondary education	51.6	25.1	7.0	86.3
% students with a foreign background	41.1	21.5	6.0	95.7

The data contained slightly fewer boys (46.5%) than girls (53.5%). About two thirds of the students lived with two parents in the same household and one third did not. One third did not have any parent with university education, whereas two thirds did. In all, 6.1% of the students reported that at least one of their parents was unemployed. With regards to migration background, 90.8% of the students had lived in Sweden for 10 years or more, and 9.2% less than 10 years. The values of school ethos varied between 34.8 and 54.3, with an average of 46.1. The study sample was divided into three groups of about equal size, distinguishing between students who attended schools with a weaker ethos (mean value 41.3; range 34.8–44.3); an intermediate level of ethos (mean value 46.0; range 44.4–48.0), and a stronger ethos (mean value 51.2, range 48.1–54.3). The school proportion of students with parents with post-secondary education varied from 7.0 to 86.3%, with an average of 51.6%. The school proportion of students with a foreign background varied between 6.0 and 95.7%, with an average of 41.1%.

Proportions of students engaged in gambling and in risk gambling, by schools’ level of teacher-rated ethos, are presented in Table 2. The percentages indicate that gambling during the past 12 months was most common among students attending schools with an intermediate level of ethos, and least common among students attending schools with a stronger ethos (χ^2^ = 43.81, *p* < 0.001). With regards to the indicators of risk gambling, a graded and consistent pattern shows that risk gambling was most common among students attending schools with a weaker ethos, and least common among students attending schools with a stronger ethos (for the overall measure of risk gambling: χ^2^ = 30.04, *p* < 0.001).

**Table 2:** Proportions of students engaged in gambling and in risk gambling, by categorical school ethos. Percent

	School ethos			
	Weak	Inter-mediate	Strong	χ^2^
Gambling				
Gambling during the past 12 months	15.2	16.8	9.3	43.81***
Risk gambling				
Tried to reduce gambling	3.4	2.0	1.0	22.66***
Felt restless or irritated when not able to gamble	2.7	1.4	1.0	16.36***
Lied about gambling	1.9	1.4	0.7	9.06*
At least one indicator of risk gambling	4.9	3.3	1.6	30.04***
n	1722	1743	1658	

***p < 0.001 **p < 0.01 *p < 0.05

To assess the associations between teacher-rated school ethos and student-reported gambling and risk gambling in a multivariate framework, a series of two-level binary logistic regression models were performed. Results from the analyses of gambling during the past 12 months are presented in Table 3. As indicated by the empty model, 7.9% of the variation in gambling could be attributed to the school level. Model 1 included student-level variables. There was a clear gender difference in that girls were less likely to have gambled than boys (OR 0.23, 95% CI 0.19–0.27). None of the other included student-level variables were statistically significantly associated with gambling, when mutually adjusted. Model 2 added the school proportion of students with parents with post-secondary education, which was negatively associated with student gambling (OR 0.99, 95% CI 0.99–1.00). Model 3 added the school proportion of students with a foreign background, which was not statistically significantly associated with gambling. Finally, categorical teacher-rated school ethos was added in Model 4. Compared with students attending a school with a relatively weak ethos, those attending schools with a strong ethos had a statistically significantly lower likelihood of having gambled during the past 12 months (OR 0.62, 95% CI 0.43–0.89). There was however no statistically significant difference in the likelihood of gambling between students in schools with a weak and an intermediate level of ethos (OR 1.02, 95% CI 0.74–1.40). We also performed analyses of the continuous measure of teacher-rated school ethos, which showed a close to statistically significant association with gambling (OR 0.86, 95% CI 0.74–1.00, *p* = 0.051) (not presented in Table).

**Table 3:** Associations between school ethos and gambling. Odds ratios from two-level binary logistic regressions. *n* = 5123 students distributed across 46 upper secondary schools

	Empty model	Model 1		Model 2		Model 3		Model 4	
		OR	95% CI	OR	95% CI	OR	95% CI	OR	95% CI
*Student level*									
Gender									
Boy (ref.)		1.00	–	1.00	–	1.00	–	1.00	–
Girl		0.23***	0.19–0.27	0.22***	0.19–0.27	0.23***	0.19–0.28	0.23***	0.19–0.28
Family structure									
Two parents in the same household (ref.)		1.00	–	1.00	–	1.00	–	1.00	–
Other		1.15	0.97–1.37	1.15	0.96–1.37	1.15	0.96–1.37	1.14	0.96–1.35
Parental university education									
No parent (ref.)		1.00	–	1.00	–	1.00	–	1.00	–
At least one parent		0.86	0.71–1.03	0.87	0.72–1.04	0.86	0.72–1.04	0.89	0.74–1.07
Parental unemployment									
No parent (ref.)		1.00	–	1.00	–	1.00	–	1.00	–
At least one parent		0.97	0.68–1.37	0.97	0.68–1.37	0.97	0.68–1.38	0.98	0.69–1.39
Migration background									
≥ 10 years in Sweden (ref.)		1.00	–	1.00	–	1.00	–	1.00	–
< 10 years in Sweden		0.79	0.57–1.07	0.81	0.59–1.10	0.81	0.60–1.11	0.80	0.58–1.09
*School level*									
% students with parents with post-secondary education				0.99*	0.99–1.00	0.99**	0.98–1.00	0.99**	0.99–1.00
% students with a foreign background						1.00	0.99–1.01	0.99	0.99–1.00
School ethos									
Weak (ref.)								1.00	–
Intermediate								1.02	0.74–1.40
Strong								0.62**	0.43–0.89
ICC	7.9%	4.8%		3.9%		3.7%		2.6%	

***p < 0.001 **p < 0.01 *p < 0.05

Results from the analyses of risk gambling are presented in Table 4. Model 1 showed substantial variation at the school level (ICC: 15.0%). In Model 1, student-level characteristics were included. A strong and clear gender difference was seen in that girls were substantially less likely than boys to have been engaged in risk gambling (OR 0.08, 95% CI 0.04–0.13). Further, students not living with two parents in the same household had a greater likelihood of risk gambling (OR 1.46, 95% CI 1.05–2.02), whereas students with at least one university-educated parent were less inclined to engage in risk gambling (OR 0.59, 95% CI 0.42–0.82). Parental unemployment and migration background were not statistically significantly associated with risk gambling, when mutually adjusting for the other student-level sociodemographic characteristics. The between-school variation in risk gambling shown by the empty model was to a great extent accounted for by student-level characteristics, as shown by the reduction of the ICC to 4.6% in Model 1. Model 2 added the school proportion of students with parents with post-secondary education and Model 3 added the school proportion of students with a foreign background. None of these control variables were however statistically significantly associated with student risk gambling. Finally, categorical teacher-rated school ethos was added in Model 4. Compared with students in schools with a weaker teacher-rated ethos, those in schools with an intermediate ethos were less likely to having engaged in risk gambling (OR 0.57, 95% CI 0.36–0.90), and those in schools with a stronger ethos to an even lesser extent (OR 0.40, 95% CI 0.22–0.69). Also analyses with the continuous school ethos measure showed a strong, statistically significant association with risk gambling, even when adjusting for student- and school-level covariates (OR 0.65, 95% CI 0.52–0.81, *p* < 0.001) (not presented in Table).

**Table 4:** Associations between school ethos and risk gambling. Odds ratios from two-level binary logistic regressions. *n* = 5123 students distributed across 46 upper secondary schools

	Empty model	Model 1		Model 2		Model 3		Model 4	
		OR	95% CI	OR	95% CI	OR	95% CI	OR	95% CI
Student level									
Gender									
Boy (ref.)		1.00	–	1.00	–	1.00	–	1.00	–
Girl		0.08***	0.04–0.13	0.08***	0.04–0.13	0.08***	0.04–0.13	0.08***	0.05–0.14
Family structure									
Two parents in the same household (ref.)		1.00	–	1.00	–	1.00	–	1.00	–
Other		1.46*	1.05–2.02	1.45*	1.05–2.01	1.45*	1.05–2.01	1.42*	1.03–1.96
Parental university education									
No parent (ref.)		1.00	–	1.00	–	1.00	–	1.00	–
At least one parent		0.59**	0.42–0.82	0.60**	0.43–0.83	0.60**	0.43–0.83	0.65*	0.47–0.90
Parental unemployment									
No parent (ref.)		1.00	–	1.00	–	1.00	–	1.00	–
At least one parent		1.22	0.69–2.17	1.23	0.69–2.17	1.23	0.70–2.18	1.27	0.72–2.24
Migration background									
≥ 10 years in Sweden (ref.)		1.00	–	1.00	–	1.00	–	1.00	–
< 10 years in Sweden		1.12	0.69–1.82	1.17	0.72–1.91	1.17	0.72–1.92	1.20	0.74–1.96
School level									
% students with parents with post-secondary education				0.99	0.99–1.00	0.99	0.98–1.00	0.99	0.98–1.00
% students with a foreign background						1.00	0.99–1.01	0.99	0.98–1.00
School ethos									
Weak (ref.)								1.00	–
Intermediate								0.57*	0.36–0.90
Strong								0.40**	0.22–0.69
ICC	15.0%	4.6%		3.4%		3.5%		1.8%	

***p < 0.001 **p < 0.01 *p < 0.05

## Discussion

Gambling is not uncommon among adolescents, and a certain minority can be classified as being engaged in problematic gambling. This calls for knowledge about risk and protective factors. Using a new, unique data material based on cross-sectional survey information collected among 1061 teachers and 5123 students in 46 upper secondary schools in Stockholm, the present study showed that teachers’ ratings of a school’s ethos was inversely associated with gambling and risk gambling among students, when adjusting for student- and school-level sociodemographic characteristics.

Overall, the results of the present study reflect previous research which has established that a strong school ethos is associated with less health risk behaviours among the students [19–21], and extend these findings by showing that such associations exist also for gambling and risk gambling. The result that high teacher ratings of a school’s ethos seem to protect against students’ inclination to engage in risk behaviours may be understood through different possible mechanisms. One such pathway may be that a strong school ethos promotes higher academic performance and motivation, leading to a lower willingness among students to involve in risk behaviours at the expense of one’s schooling and future career. Another pathway may be that stronger school ethos is associated with higher perceived teacher caring among the students [23]. It is possible that students who feel that they receive a great amount of social support and respect, and perhaps also see their teachers as role models, are less likely to want to engage in risk behaviours compared with students who do not experience such beneficial relations with their teachers. Indeed, positive student-teacher relationships have been shown to be negatively associated with student substance use [24]. Yet another pathway could be students’ future orientation. A previous study which was based on the same data material as the current study reported that a strong school ethos, as rated by teachers, was linked with an optimistic future orientation among the students [25]. A pessimistic future orientation, by contrast, has been connected with a greater likelihood of engaging in risk gambling [27] as well as other health risk behaviours [26]. The interpretation is that individuals who sense a lack of future prospects are more inclined to seek immediate benefits rather than to invest in behaviour associated with delayed gratification, which make them more prone to involve in risk behaviours [26].

These potential mechanisms may affect individual students’ behaviour directly, but it is also possible that they operate through the behaviours of peers, which in turn influence individual behaviour. To gain a deeper understanding of the mechanisms through which school ethos may affect student outcomes, a promising task for future research would be to explore possible pathways using statistical techniques such as structural equation modelling, but also to apply qualitative methods such as personal interviews and focus groups among students.

The main strength of the study is the data material used, with survey information collected among teachers and students in the same schools. The use of different data sources limits the risk of common methods variance. Another advantage is the fact that the data contains a relatively large number of students since this allows analyses of risk gambling, which is, after all, confined to a minority of students. The relatively high response rate among students (78%), due to the fact that the data were collected in classrooms, is also a strength. Nevertheless, the study is not without limitations. One weakness is the fact that our measures of gambling and of risk gambling are not based on established scales, although the measure of risk gambling has been shown to have good validity [30]. Yet, the percentages reporting to be involved in gambling and in risk gambling largely reflect those reported in studies that use other measures [4, 32]. Also our measure of teacher-rated school ethos was not extracted from a pre-existing scale but was developed for this particular data collection, which may have limited the validity. Yet, the items were theory-based and formulated with the explicit purpose of capturing school ethos, and empirically, the index showed good psychometric properties [29]. Another limitation is the fact the data are cross-sectional and hence we cannot draw the conclusion that there is a causal effect of school ethos on adolescents’ inclination to engage in gambling and in risk gambling. The response rate among teachers (58%) was lower than that of students. It is likely that there was systematic attrition in that the teachers who were most stressed (and least satisfied with their work environment) were less inclined to participate. This may have implied an overestimation of the overall level of school ethos in the data. However, we do not see any reasons that this possible bias would have affected the associations between teacher-rated school ethos and student gambling to any substantial degree. Furthermore, it cannot be ruled out that there are omitted variables that are associated with both teachers’ ratings of the school ethos and with student risk gambling, that may affect the associations found. For instance, we cannot empirically solve the issue of selection. It is indeed possible that students with certain characteristics are more likely to be enrolled in schools with higher teacher ratings of ethos and also less inclined to engage in gambling and in risk gambling. Yet, in an attempt to isolate the associations of interest as much as possible, we controlled for sociodemographic characteristics at both the student- and the school-level.

## Conclusions

The current study indicated that a strong school ethos may contribute to prevent against students’ inclination to engage in gambling and in risk gambling. In more general terms, the study provides evidence that schools’ values and norms as reflected in their teacher-rated ethos have the potential to counteract unwanted behaviours among the students, and accordingly, that improving a school’s ethos may promote positive development among youth.

## Data Availability

The data are not publicly available. Access to data from the Stockholm School Survey can be applied for at Stockholm Municipality, Sweden. Access to data from the Stockholm Teacher Survey can be applied for at the Department of Public Health Sciences, Stockholm University, Sweden.

## Abbreviations

95% CI: 95% Confidence Interval
ADHD: Attention Deficit Hyperactivity Disorder
ESPAD: European School Survey Project on Alcohol and Other Drugs
ICC: Intraclass Correlation
OR: Odds Ratio
SSS: Stockholm School Survey
STS: Stockholm Teacher Survey
